# Evaluation of the acoustic coordinated reset (CR ®) neuromodulation therapy for tinnitus: study protocol for a double-blind randomized placebo-controlled trial

**DOI:** 10.1186/1745-6215-14-207

**Published:** 2013-07-10

**Authors:** Derek J Hoare, Robert H Pierzycki, Holly Thomas, David McAlpine, Deborah A Hall

**Affiliations:** 1 National Institute for Health Research (NIHR) Nottingham Hearing Biomedical Research Unit, University of Nottingham, Ropewalk House, 113 The Ropewalk, Nottingham NG1 5DU, UK; 2 University College London (UCL) Ear Institute, 332 Gray’s Inn Road, London WC1X 8EE, UK

**Keywords:** Tinnitus, Pathological synchrony, Sound therapy

## Abstract

**Background:**

Current theories of tinnitus assume that the phantom sound is generated either through increased spontaneous activity of neurons in the auditory brain, or through pathological temporal firing patterns of the spontaneous neuronal discharge, or a combination of both factors. With this in mind, Tass and colleagues recently tested a number of temporally patterned acoustic stimulation strategies in a proof of concept study. Potential therapeutic sound regimes were derived according to a paradigm assumed to disrupt hypersynchronous neuronal activity, and promote plasticity mechanisms that stabilize a state of asynchronous spontaneous activity. This would correspond to a permanent reduction of tinnitus. The proof of concept study, conducted in Germany, confirmed the safety of the acoustic stimuli for use in tinnitus, and exploratory results indicated modulation of tinnitus-related pathological synchronous activity with potential therapeutic benefit. The most effective stimulation paradigm is now in clinical use as a sound therapy device, the acoustic coordinated reset (CR®) neuromodulation (Adaptive Neuromodulation GmbH (ANM), Köln, Germany).

**Methods/Design:**

To measure the efficacy of CR® neuromodulation, we devised a powered, two-center, randomized controlled trial (RCT) compliant with the reporting standards defined in the Consolidated Standards of Reporting Trials (CONSORT) Statement. The RCT design also addresses the recent call for international standards within the tinnitus community for high-quality clinical trials. The design uses a between-subjects comparison with minimized allocation of participants to treatment and placebo groups. A minimization approach was selected to ensure that the two groups are balanced with respect to age, gender, hearing, and baseline tinnitus severity. The protocol ensures double blinding, with crossover of the placebo group to receive the proprietary intervention after 12 weeks. The primary endpoints are the pre- and post-treatment measures that provide the primary measures of efficacy, namely a validated and sensitive questionnaire measure of the functional impact of tinnitus. The trial is also designed to capture secondary changes in tinnitus handicap, quality (pitch, loudness, bandwidth), and changes in tinnitus-related pathological synchronous brain activity using electroencephalography (EEG).

**Discussion:**

This RCT was designed to provide a confident high-level estimate of the efficacy of sound therapy using CR® neuromodulation compared to a well-matched placebo intervention, and uniquely in terms of sound therapy, examine the physiological effects of the intervention against its putative mechanism of action.

**Trial registration:**

ClinicalTrials.gov, NCT01541969

## Background

The phantom auditory sensation of tinnitus is transiently experienced by most people, but for 10 to 15% of the population, and up to one in three of older adults, the experience is chronic [1,2]. Although factors contributing to tinnitus potentially include otologic, neurologic, infectious, and drug-related effects, it is most readily associated with noise exposure and aging [3-5]. Among the most frequently reported difficulties associated with tinnitus are sleep disturbance, hearing difficulties, social withdrawal, and negative emotional reactions, such as anxiety and depression [6-10]. Intrusive tinnitus is, therefore, a complex condition that arises with different etiologies and comorbidities. Management of tinnitus typically involves relieving the distress, anxiety or depression that can accompany tinnitus, masking the sound by introducing external sound, or reducing the neural signal believed to be causing the tinnitus.

Current theories of tinnitus assume that the phantom sound is generated either through increased spontaneous activity of neurons in the auditory brain, or through pathological synchrony of the spontaneous neuronal discharge, or a combination of both factors (see Roberts *et al*. for a review [11]). Theoretical modeling studies indicate that such aberrant activity patterns might be reduced through acoustic stimulation [12,13]. While Schaette and Kempter [12] proposed that sound enrichment provided by prolonged use of a hearing aid could counteract neuronal hyperactivity and reduce tinnitus, Tass and Popovych [13] proposed using sound to interrupt pathological connectivity and synchronous firing that may underlie tinnitus in a process termed ‘coordinated reset’ (CR®). CR® was first modeled computationally by Tass [14] as a method using high-frequency pulse trains to desynchronize the concerted activity of neural subpopulations. Tass *et al*. [15] later generated evidence for applications in the domain of deep brain electrical stimulation for neurological diseases, such as Parkinson’s disease, using an animal model. Given that the model of CR® was not specific to electrical stimuli, Tass and Popovych [13] also modeled the potential CR® effects of acoustic stimulation on tinnitus-related brain activity, concluding it to hold promise as a tinnitus treatment. Tass *et al*. [16] recently conducted a proof of concept study on humans testing acoustic CR® algorithms predicted to disrupt hypersynchronous neuronal activity, and to promote plasticity that stabilizes a state of asynchronous spontaneous activity. Such an effect would correspond to a permanent reduction of tinnitus [13]. The most effective stimulus paradigm is now in clinical use as a sound therapy device, the acoustic CR® neuromodulation (Adaptive Neuromodulation GmbH (ANM), Köln, Germany). While the proof of concept study conducted by Tass *et al*. [16] confirmed the safety of the sound stimulus, it also provided exploratory level evidence that CR® neuromodulation may be efficacious, in terms of reducing the reaction to tinnitus and modulating tinnitus-related pathological synchronous activity. However, a Phase 2 clinical trial is now required to determine efficacy in a controlled and powered sample.

### Purpose

This Phase 2 randomized controlled trial (RCT) will determine whether the algorithmic auditory stimulation delivered by the CR® neuromodulation device has significant benefit over a placebo stimulus delivered by the same device.

### Primary objectives

• Does CR® neuromodulation significantly reduce tinnitus intrusiveness compared to an active placebo control?

### Secondary objectives

• Does CR® neuromodulation significantly change the percept of tinnitus compared to an active placebo control?

• Does CR® neuromodulation significantly alter neural temporal firing patterns, as measured by spontaneous low-frequency oscillatory responses in the brain (electroencephalography (EEG)), compared to the placebo control?

## Methods/Design

**Figure 1:**
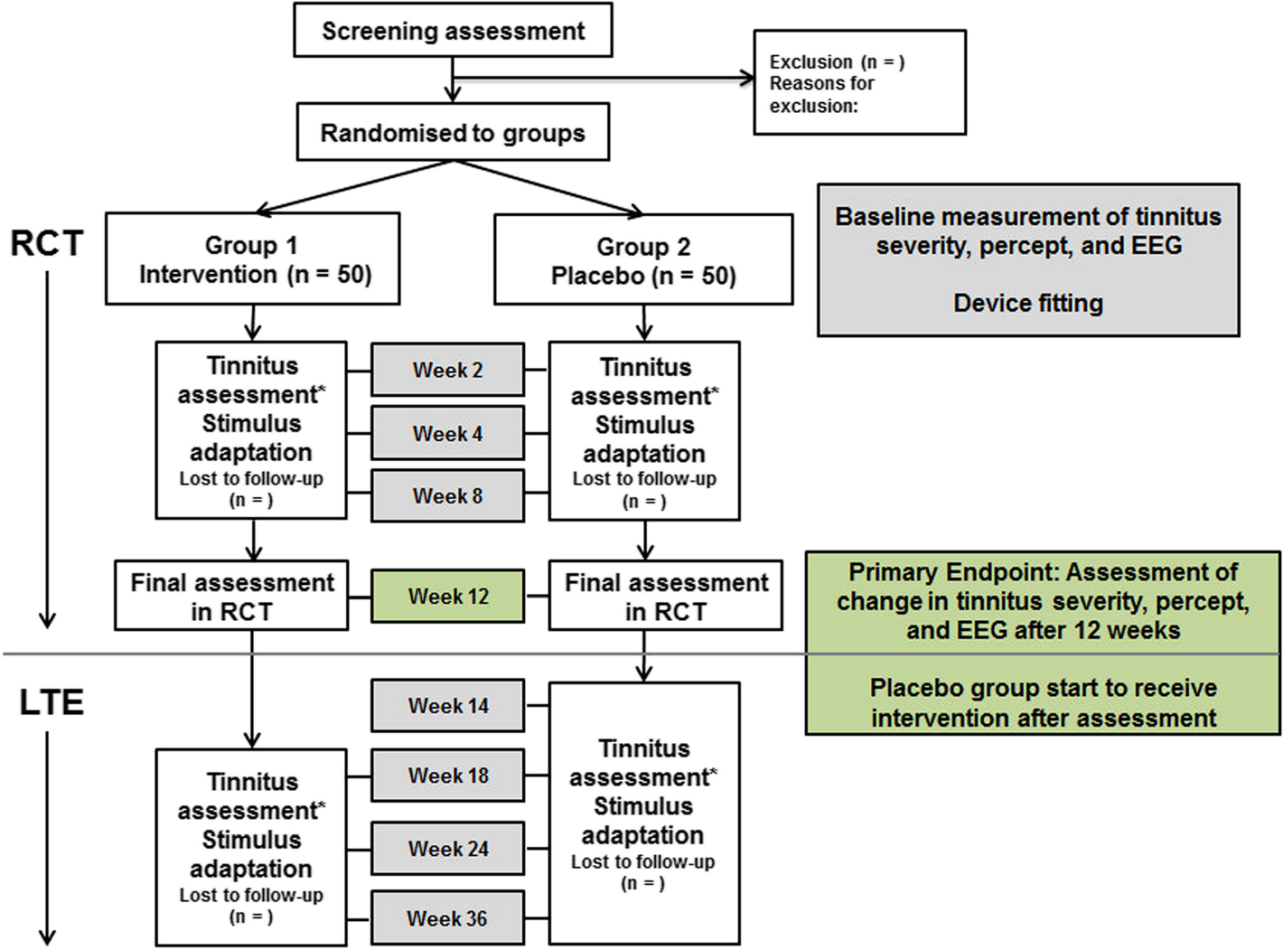
**Trial flow chart.** *Tinnitus assessment will include psychoacoustic (Tinnitus Tester) measures and questionnaire measures in the RCT phase. Tinnitus Tester measures will not be taken in the LTE phase. EEG, electroencephalography; LTE, long-term extension; RCT, randomized controlled trial.

This is a two-center, double-blind, placebo-controlled study with minimized allocation of participants to one of two groups. Center 1 is the National Institute for Health Research (NIHR) Nottingham Hearing Biomedical Research Unit, Nottingham, UK. Center 2 is the University College London (UCL) Ear Institute, London, UK. Participants meeting the inclusion criteria will be randomized to either the intervention group (Group 1) or the placebo group (Group 2) of a 12-week RCT. After 12 weeks, the placebo group will be unblinded and continue on the study according to the treatment protocol. After the RCT, all participants will be followed for a further 24 weeks in a long-term extension (LTE) arm (Figure 1). The primary endpoints are the pre- and post-intervention metrics that provide the primary measures of efficacy, that is, responses about thoughts and feelings associated with tinnitus measured using a validated questionnaire. Repeated measures of general quality of life, tinnitus handicap (as measured by two other tinnitus questionnaires), resting state EEG, and psychoacoustic measure of tinnitus percept will also be collected as secondary outcome measures.

Permission to conduct the study was granted by the National Research Ethics Service (NRES) Committee, East Midlands – Nottingham 1, Nottingham, UK, and the trial sponsor is the Nottingham University Hospitals National Health Service (NHS) Trust, Research and Innovation department, Nottingham, UK.

### Population and sample size

Participants will be recruited through leaflets placed in audiology, and ear, nose and throat (ENT) departments, and any direct contacts made to the recruiting centers in response to publicity in the national media. Written informed consent will be obtained from each participant in accordance with the permissions granted. The primary outcome measure of treatment efficacy is the Tinnitus Handicap Questionnaire (THQ) [17], where the maximum score is 2,700. THQ data from a published study of tinnitus maskers were used to estimate the required sample size (two-sample *t*-test power analysis, performed in *R*). After a 12-week intervention, Henry *et al*. [18] found that a difference in mean THQ score of 194 between groups with a pooled standard deviation (SD) of 450 was considered significant, and represented a medium effect size. If we therefore assume a difference in mean THQ score of 250 between groups with a pooled SD of 425 as representative of a large effect size, then for a two-sided significance level of 0.05 and 80% power, it is estimated that 47 participants are required for each group. We assume a low drop-out rate of approximately 5% in 12 weeks and therefore a total of 100 participants will be recruited.

#### Inclusion criteria

• Men and women ≥18 years of age

• Pure-tone average (PTA) hearing thresholds <60 dB HL (0.5, 1, 2, 4 kHz) in the ear where tinnitus is perceived

• Must be able to hear stimulation tones presented by the device at all frequencies

• Chronic subjective tinnitus for more than 3 months

• Dominant tinnitus frequency measured between 0.2 and 10 kHz

• At least mild tinnitus, score ≥18 on Tinnitus Handicap Inventory (THI) [19]

• Willing to wear the device for 4 to 6 hours daily during the trial

• Sufficient command of English language to read, understand and complete the questionnaires

• Able and willing to give informed consent

#### Exclusion criteria

• Objective tinnitus, Ménière’s disease, temporomandibular joint disorder

• Pulsatile tinnitus

• Intermittent tinnitus

• Severe anxiety, >25 score on the Beck Anxiety Inventory (BAI) [20]

• Severe depression, >29 score on the Beck Depression Inventory (BDI-II) [20]

• Catastrophic tinnitus, score ≥78 on the THI

• Hearing aid wearers for less than 9 months, or long-term hearing aid wearers who have had prescription adjustments within last 3 months

• Pure-tone absolute hearing thresholds >70 dB on individual frequencies up to 8 kHz (unable to sufficiently hear the stimulus)

• Taking part in another trial during the 30 days before study start

• The individually tailored training stimulus is uncomfortable or not acceptable to the participant

Pure-tone audiometry will be conducted in a soundproof booth using the Unity 2 system (Siemens, Berlin, Germany) and HDA 200 headphones (Sennheiser, Wedemark, Germany), measuring hearing thresholds between 125 and 12,000 Hz.

Participants will be withdrawn from the trial at any point if they commence any other form of tinnitus therapy, or if following commencement of the study they report an adverse event. The definition of an adverse event in the context of this study is either: 1) since wearing the device tinnitus intrusiveness increases and makes it unbearable; or 2) a disease or symptom (unrelated to tinnitus) present at baseline worsens following the start of the study.

### Randomization and blinding

Randomized allocation to groups will be based on a minimization protocol (Minim) [21] and conducted by a researcher who is independent of the trial. Minimization will be used here to match groups for age: 18 to 49 years, 50 to 69 years, 70 or over years (categories based on statistics for moderate to severe hearing loss sourced from the Royal National Institute for Deaf People, now Action on Hearing Loss [22]), gender, hearing loss (PTA at 0.5, 1, 2, 4 kHz), and grade of tinnitus severity (using THI categories defined in [23]).

Participants will be randomly assigned initially to receive the treatment (Group 1) or placebo (Group 2) stimulation. After 12 weeks, participants in the placebo group will crossover to start receiving the treatment according to the intervention algorithm, and will follow the same protocol as initially delivered to Group 1.

The RCT phase of this study will be conducted double-blind. Participants will not know the group to which they are allocated (intervention or placebo). The researcher assessing outcomes will not know which group the participant was allocated to. The audiologists fitting and adjusting the device will be blind to group allocation since they will be unaware whether the device programming code supplied to them after the participant is randomized generates an intervention or placebo stimulus. All participants will receive the same information about the expected trial outcomes.

### Intervention

**Figure 2:**
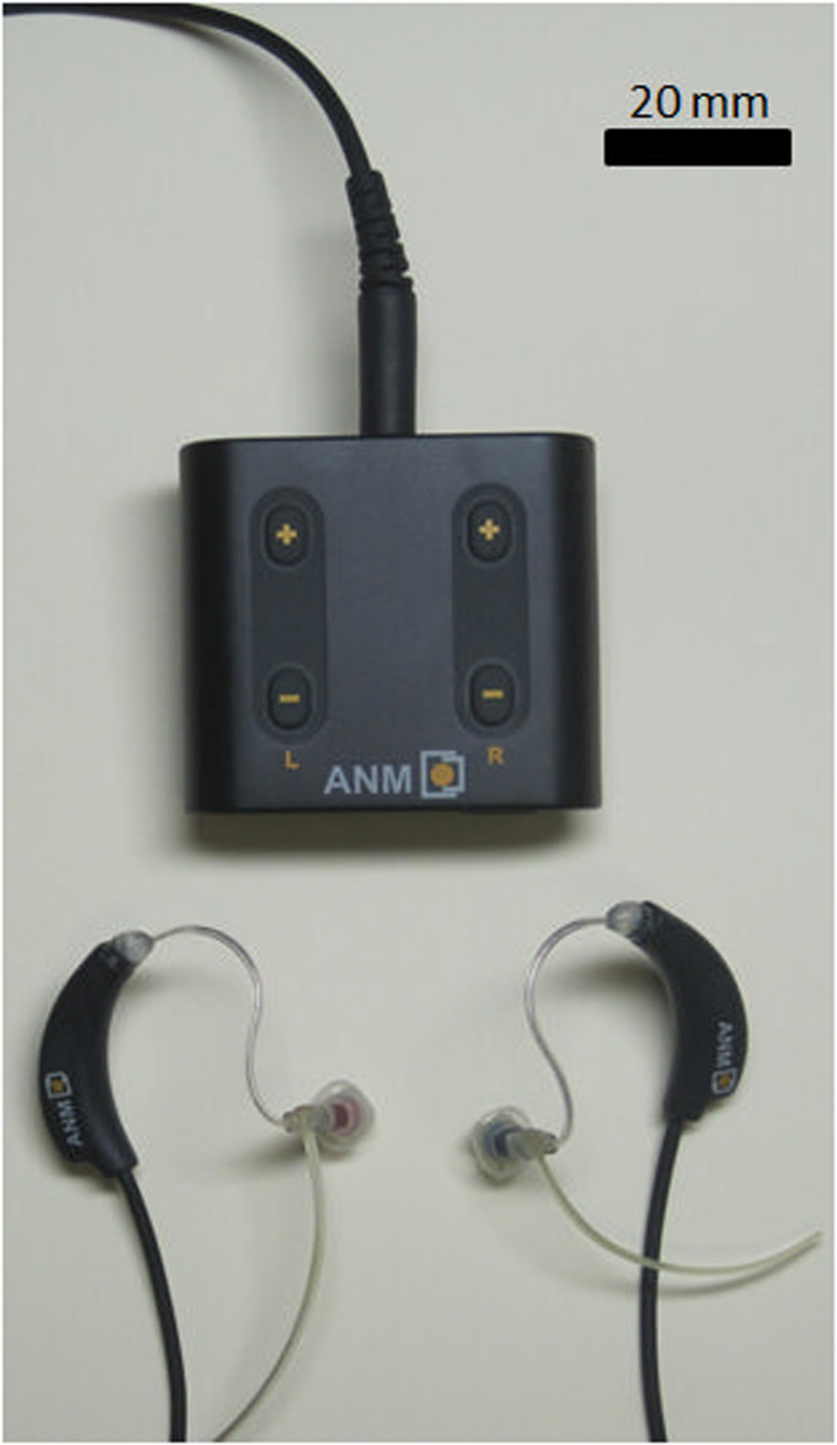
**Image of the acoustic CR® neuromodulation device.** CR, coordinated reset.

The CR® neuromodulation device is small, lightweight, and connected to a pair of custom in-ear earphones (Figure 2). The device will be fitted by an appropriately trained audiologist using a custom sound console to determine a pitch matching the participant’s tinnitus percept, and to calculate a unique sequence of tones around that pitch to be employed in the treatment protocol for each individual. The tones are presented by the device at a level that is only slightly higher than the hearing threshold for those tones, that is, a comfortable quiet level that should not interrupt the participant’s normal daily routine. Participants will be instructed to wear the device for 4 to 6 hours daily during the RCT stage. During the LTE stage, participants in the intervention group will be advised to wear the device for at least 4 hours daily.

### Placebo control

Participants in the placebo group will also receive the same device and be presented with a sequence of tones. The placebo tones will include frequencies in the 500 to 4,000 Hz range, but excluding frequencies around the tinnitus pitch. The placebo tones will also be delivered at a slower repetition rate and are predicted not to have the same therapeutic effect as the treatment stimulus. The placebo does, however, represent an active intervention, since the participant may experience some level of tinnitus masking while the device is worn.

### Device fitting procedure

The tinnitus assessment and the device programming procedure will be identical for all participants. The individual’s dominant tinnitus pitch is determined using an adaptive bracketing method. To first establish a ‘bracket’, pure tones from 500 to 12,000 Hz are presented to the individual in an ascending/descending sweep with a step size of 500 Hz, and participants select tones that border similarity to their tinnitus percept. Once this bracket has been established, a two-alternate forced-choice method is used to determine a dominant tinnitus pitch. Once a dominant pitch is reliably identified, the proprietary software uses an algorithm to program a set of four stimulation tones that span above and below the tinnitus pitch. Tones are then loudness matched, such that the participant subjectively equates each tone to be at a soft but audible listening level. All tones are compared against each other to ensure equal loudness.

**Figure 3:**
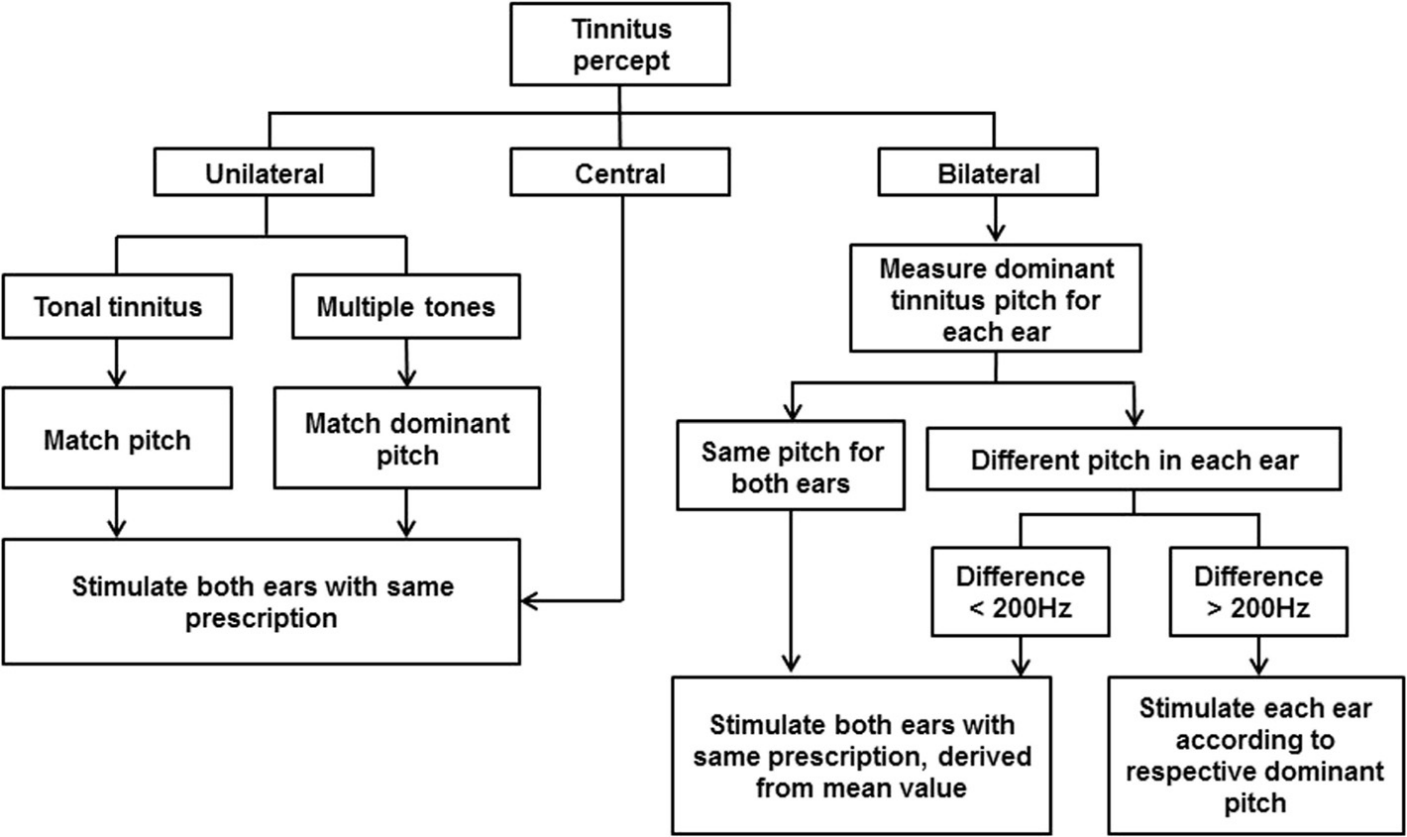
**Device fitting protocol for differences in tinnitus percept between ears.** If tinnitus is identified as central (not in the ear) then only one ear need be assessed.

All participants are prescribed with bilateral stimulation. Participants with unilateral tinnitus (experienced in one ear) will be prescribed bilateral stimulation with the same prescription. Participants with bilateral tinnitus that differs in pitch between ears by more than 200 Hz will require different stimulation prescriptions for each ear. All participants who do not perceive tinnitus to be in the ear will be prescribed bilateral stimulation with the same prescription (Figure 3).

Participants will be reassessed at planned intervals over the 36 weeks of the trial (Figure 1), or at an interim interval if they experience a noticeable change in tinnitus pitch. The device prescription will be reassessed at each visit, and when there is a change in tinnitus pitch it will be adjusted accordingly. When a participant’s tinnitus pitch is increased beyond the output limits of the device, that is, tinnitus at or above 10.5 kHz, the participant will pause using the device for 2 weeks, and will then be reassessed. If at this assessment tinnitus remains at or above 10.5 kHz, the participant will be withdrawn from the trial.

### Primary outcome measure

The primary measure of efficacy will be change in THQ score between baseline and week 12. At the time of writing the protocol for the funding application, this questionnaire was one of the better validated measures of tinnitus severity, responsive to treatment-related change, and with a test-retest reliability of 0.93 [24]. The sample size is powered according to previous trial data using this measure [18].

### Secondary outcome measures

#### Questionnaires

The World Health Organization (WHO) Quality Of Life-BREF (WHOQOL-BREF) is a 26-item questionnaire measuring self-perceived quality of life in four domains (physical health, psychological, social relationships, environment) with test-retest reliability of 0.66 to 0.80 [25].

In the UK, the THI is the most common clinical tool used for diagnosis, and sometimes outcome assessment [26]. It is a 25-item measure of tinnitus severity with test-retest reliability of 0.93.

We will also add the Tinnitus Functional Index (TFI) questionnaire [27] to the assessment battery. This 25-item questionnaire was very recently developed as a sensitive measure of treatment-related change in tinnitus distress with a clinically significant change defined at 13 points. It is also a diagnostic tool for tinnitus severity with high test-retest reliability of 0.97.

Participants will also be provided with a paper diary and asked to log their daily use of the device, and note any experience or comment on its usability.

#### Psychoacoustic measures of tinnitus loudness and pitch

In addition to the pitch match performed as part of the device fitting procedure, baseline and changes in tinnitus quality (loudness, bandwidth, dominant pitch) will also be assessed using the Tinnitus Tester developed by Roberts *et al*. [11]. For loudness matching, participants adjust the level of 11 sound clips (center frequencies 500 to 12,000 Hz) until each one is perceived to equal that of the tinnitus sound. A sensation level is then calculated according to the matched value at a single frequency where there is little or no hearing loss and the frequency is distant from the dominant tinnitus pitch. Next, a tinnitus spectrum representing bandwidth is generated by asking participants to rate the likeness of the same 11 sounds to the pitch of their tinnitus, using a 100-point scale. Bandwidth can then be calculated as the SD of all frequencies in the tinnitus spectrum, where each frequency is weighted by its percentage likeness to the tinnitus pitch identified by the participant (compare [28]). A dominant tinnitus pitch is taken as the frequency in the spectrum with the highest likeness rating.

#### EEG spontaneous oscillatory activity

To assess changes in spontaneous oscillatory activity, EEG will be performed using a Neuroscan system (SynAmps^2^ model 8050, Compumedics Neuroscan, Charlotte, NC, USA) with 66 equidistant scalp electrodes. EEG will be conducted at baseline and at the end of the 12-week RCT for the first 50 participants (25 intervention, 25 placebo) attending Center 1 in Nottingham. At the 12-week visit, EEG will be performed after behavioral assessment, at a minimum 2-hour break from stimulation. For the recording, participants will be seated in a quiet, darkened soundproof booth, have their eyes open and be instructed to fix their gaze on a marker point in front of them. A central frontal electrode will be used as ground and a nose-tip electrode as reference, and two additional electrodes will be placed below the eyes to monitor eye movements. Electrode impedances will be maintained below 5 kΩ prior to the start of the recordings. EEG recordings will be collected over a continuous 10-minute period with a 0.5 to 200 Hz passband, and digitized at a 1,000 Hz sampling rate.

### Statistical methods, data analysis and reporting

Analysis will be performed to include all 100 participants who meet the study criteria and are fitted with the device. Participants leaving the study before its completion will not be replaced. Any missing data will be imputed using an expectation-maximization method, which assumes a normal distribution for the partially missing data and bases inferences on the likelihood under that distribution (maximum 25 iterations, performed in SPSS v16.0, IBM, Armonk, USA). Effect sizes will be calculated with Cohen’s *d* computed with the pooled SD of the two groups.

Following completion of the trial, the sponsor will use all reasonable endeavors to ensure the appropriate publication of the research. The manufacturer’s approval of the manuscript will be sought prior to submission. However, the investigators have the right to publish results of the statistical analysis, conducted according to the protocol, whether they are positive or negative, and whether they support or negate the commercial interests of the manufacturer.

Baseline characteristics of participants will be reported as descriptive statistics, including but not limited to age, gender, hearing levels, anxiety, depression, medication, tinnitus duration, tinnitus severity, tinnitus dominant pitch, and tinnitus loudness estimates. Comparisons between groups will assess the degree to which comparability of randomization was achieved.

### Efficacy analyses

Our primary endpoint and first analysis of data will be the end of the 12-week RCT. Analysis will involve a summary of within- and between-group comparisons with respect to the primary and secondary outcome measures detailed above using paired *t*-test/McNemar’s test, *t*-test, and analysis of variance (ANOVA)/Kruskal-Wallis test analyses, as appropriate. In addition, (the simple and/or multiple) linear regression/general linear model/generalized linear model approaches will be performed, adjusting for the factors employed in minimizing participants to groups, as appropriate.

The second endpoint and planned analysis will be at the 24-week stage to examine the effect of 12 weeks’ intervention across all 100 participants. The final analysis of long-term effects will examine all 100 participants at trial completion (36 weeks).

Subgroup analyses will be performed to identify a participant population that demonstrates the most clinically significant improvement.

EEG recordings (only performed on half of the participants) will be analyzed using EEGLAB [29], an open source toolbox for advanced EEG analysis (Swartz Center for Computational Neuroscience (SCCN), University of California San Diego, CA, USA) run under MATLAB (The Mathworks, Natick, MA, USA). Pre-processed data (high- and low-pass filtering, offline epoching, artifact correction) will be further corrected for artifacts using independent component analysis (ICA) in EEGLAB [30]. Power analysis will be carried out in the EEGLAB, and obtained power spectra will be divided to analyze oscillatory activity in normalized EEG frequency bands. Analysis of sources of spontaneous oscillatory activity will also be conducted in EEGLAB (and/or standardized low resolution brain electromagnetic tomography (sLORETA)). Non-parametric statistical tests will be used to test for significant differences in any change in oscillatory activity between the placebo and intervention group. Within-group comparisons of activity recorded pre- and post-intervention will also be performed.

### Safety analysis

Adverse events that occur during the trial will be recorded and reported according to the trial sponsor’s standard operating procedure (SOP) for medical device trials (Nottingham University Hospitals NHS Trust SOP 52).

## Discussion

Various forms of sound and sound enrichment are in use for the clinical management of tinnitus. Typically sound is used to either mask tinnitus (introduce enough sound energy to cover up the percept of tinnitus and provide a temporary relief), partially mask tinnitus (reduce the percept so that the patient can adjust or ‘habituate’ to the sound), or to promote relaxation or distract attention away from the tinnitus sound [31]. Such approaches clearly target the psychological component of tinnitus (the negative emotional reaction), but are not clearly linked to any putative physiological mechanism of tinnitus generation. Acoustic CR® neuromodulation, however, explicitly targets a physiological marker of tinnitus, that of pathological synchronous oscillatory activity in the brain [32-35], measurable using EEG.

To generate high-level evidence for the efficacy of CR® neuromodulation, the RCT described here was designed to meet the reporting standards defined in the Consolidated Standards of Reporting Trials (CONSORT) Statement [36]. The RCT design also addresses the recent call for an international standard within the tinnitus community [37] in the form of a powered, blinded RCT with a mix of meaningful and validated outcome measures, to best capture clinical significance and change in tinnitus percept and related physiology.

## Trial status

The trial is currently in recruitment phase.

## Abbreviations

ANOVA: Analysis of variance; BAI: Beck anxiety inventory; BDI-II: Beck depression inventory; CONSORT: Consolidated standards of reporting trials; CR: Coordinated reset; EEG: Electroencephalography; ENT: Ear, nose and throat; ICA: Independent component analysis; LTE: Long-term extension; NHS: National Health Service; NIHR: National Institute for Health Research; NRES: National Research Ethics Service; PTA: Pure-tone average; RCT: Randomized controlled trial; SCCN: Swartz Center for Computational Neuroscience; SD: Standard deviation; sLORETA: Standardized low resolution brain electromagnetic tomography; SOP: Standard operating procedure; TFI: Tinnitus functional index; THI: Tinnitus handicap inventory; THQ: Tinnitus handicap questionnaire; UCL: University College London; WHO: World Health Organization; WHOQOL-BREF: WHO quality of Life-BREF.

## Competing interests

DJH, DM and DAH were awarded industry grants from The Tinnitus Clinic (Brook Henderson Group, Reading, UK), and Adaptive Neuromodulation GmbH (ANM), Köln, Germany) to conduct this trial. RHP and HT are employed on this funding.

## Authors’ contributions

DJH, DM and DAH developed the protocol. DJH, RHP, HT and DM drafted the manuscript. All authors contributed to final editing of the manuscript, and read and approved the final manuscript.
